# Supplementary material for: Asynchronous magnetic resonance elastography: Shear wave speed reconstruction using noise correlation of incoherent waves
Source: Magn Reson Med. 2022 Oct 27;89(3):990–1001. doi: 10.1002/mrm.29502 (PMC9792433; doi:10.1002/mrm.29502)
Supplement: Supplementary file 1 — DATA S1 MATLAB code used to generate the numerical simulations of Figure 1 [file MRM-89-990-s002.zip › k-Wave/helpfiles/getComputerInfo.html]

getComputerInfo :: Functions (k-Wave)


# getComputerInfo

Return information about computer and k-Wave version.

## Syntax

```
comp_info = getComputerInfo()
```

## Description

`getComputerInfo` returns information about the computer currently being used, including the versions of MATLAB and k-Wave.

## Outputs

|  |  |
| --- | --- |
| `comp_info` | structure with the following fields:   `.date`  `.computer_name`  `.operating_system_type`  `.operating_system`  `.user_name`  `.matlab_version`  `.kwave_version`  `.kwave_path` |

## See Also

`ver`
